# Language and Visual Perception Associations: Meta-Analytic Connectivity Modeling of Brodmann Area 37

**DOI:** 10.1155/2015/565871

**Published:** 2015-01-12

**Authors:** Alfredo Ardila, Byron Bernal, Monica Rosselli

**Affiliations:** ^1^Department of Communication Sciences and Disorders, Florida International University, Miami, FL 33199, USA; ^2^Radiology Department and Research Institute, Miami Children's Hospital, Miami, FL 33155, USA; ^3^Department of Psychology, Florida Atlantic University, Davie, FL 33314, USA

## Abstract

*Background.* Understanding the functions of different brain areas has represented a major endeavor of neurosciences. Historically, brain functions have been associated with specific cortical brain areas; however, modern neuroimaging developments suggest cognitive functions are associated to networks rather than to areas. *Objectives.* The purpose of this paper was to analyze the connectivity of Brodmann area (BA) 37 (posterior, inferior, and temporal/fusiform gyrus) in relation to (1) language and (2) visual processing. *Methods.* Two meta-analyses were initially conducted (first level analysis). The first one was intended to assess the language network in which BA37 is involved. The second one was intended to assess the visual perception network. A third meta-analysis (second level analysis) was then performed to assess contrasts and convergence between the two cognitive domains (language and visual perception). The DataBase of Brainmap was used. *Results.* Our results support the role of BA37 in language but by means of a distinct network from the network that supports its second most important function: visual perception. *Conclusion.* It was concluded that left BA37 is a common node of two distinct networks—visual recognition (perception) and semantic language functions.

## 1. Introduction

Understanding the specific function of different brain areas has represented one of the major challenges of neurosciences. Based on the correlation between neurological deficits and cerebral postmortem findings, the nineteen-century neuroanatomists developed the brain function localization model. Brodmann gave a strong support to this model in 1909 [1] by describing 52 pairs of brain cortical areas characterized by different laminar organization. Despite the subsequent development of more detailed cortical maps such as the map published by Economo and Koskinas [2], the so-called “Brodmann areas” (BA) have become broadly used in contemporary neuroanatomy providing a topographical substrate to specific brain functions.

Traditional models of language (e.g., [3–5]) consider language in terms of specific brain areas (such as Broca's and Wernicke's area) devoted to specific functions (e.g., language production and language understanding); contemporary neuroscience, however, emphasizes the idea of brain systems including the traditional language areas as well as other areas which could not be exclusively associated to language but are involved in language processing (e.g., [6]), such as BA37.

During the last decades the concept of “connectome” has become particularly important in understanding the brain systems of interconnections [7, 8]. The term “connectome” was introduced in 2005 to refer to the comprehensive description of the brain connections among the different areas provided by cortical parcellation maps [9]. Parcellation maps may vary in number of discrete independent areas (e.g., by gyri, cytoarchitectonic criteria as proposed by Brodmann [1] or C. Vogt and O. Vogt [10]). As a result the connectome dimension may also vary. The brain connectome may be represented as a matrix [9], as a complex graph of nodes and edges [11], or as a collection of fiber tracts derived from diffusion tensor imaging tractography [12]. These models parcel the brain in a new manner. An intricate collection of volumes, virtual shapes, and geometries arises, giving ground to paths for data flow through different processing centers. Here resides the importance of describing brain connectivity.

Several BA have been extensively studied while others have received less attention. Language areas are probably the most frequently analyzed. Language production is related to the activity of BA44 and BA45, also known as Broca's area [13–21]. Of note is that whereas Broca's area is a well delimited anatomical region encompassing the* pars triangularis* and* opercularis* of the left inferior frontal gyrus, the extension and limits of the receptive language area or Wernicke's area are not so well defined. Usually it is accepted that Wernicke's area includes BA22 and probably BA21 [22, 23]. However, several neuroimaging studies have reported left BA37 activation associated with different language tasks (see [24]). Different reports support the involvement of BA37 in semantic categorization, that is, using a particular word to refer to a collection of objects (i.e., many different objects can be named with the single word “chair,” because they have certain commonality) (e.g., [5, 25]). Finding and producing words (word retrieval and word generation) are potential functions of BA37 considering that the destruction of this area is associated with word selection anomia [16, 20]; some studies indeed have found that word search results in an increased activity in this area [26, 27]. Moreover, the involvement of BA37 in naming has been well documented [21, 28–34].

The functions of this area are not limited to oral language but extend to written language as well. The activation of BA37 has been reported during reading, taking into account that left occipital-temporal lesions are strongly associated with alexia [35–39]. The function of linking orthography to phonology reported by Hashimoto and Sakai [40] consequently points to the seemingly core function of BA37 area: associating visual information (orthography—written language—or whatever visual representation) with auditory linguistic information (phonology or spoken words). Therefore, BA37 has a visual perceptual function as well as an auditory linguistic function. Despite all these oral and written language functions, it is not a pure language area. It is also involved in visual processing [41, 42] as part of the* ventral stream*, or* what system*, which also includes the occipital striate (BA17) and prestriate (BA18 and BA19) areas.

The anatomical and clinical evidence of the visual and auditory processes mediated by BA37 suggest that this area does not work in isolation, but rather it may be part of multiple neural networks [43, 44]. To improve our understanding on how this region interacts with other brain regions we used a recent application of neuroimaging methods to develop a connectivity model of this brain area. Knowing these cerebral interactions has the potential to significantly advance our understanding of brain organization of healthy and deviant language and visual processing. The association between discrete cortical areas and specific cognitive functions has been useful in explaining clinical syndromes and in understanding brain organization underlying cognitive processes. However, the observation that most of the BA, including BA37, seems to participate in more than one specific function suggests a need for further explanatory models. That is why modern neuroimaging studies have tackled this issue focusing on linking function to networks, rather than to specific brain areas. Noteworthy, BA37 has not overt anatomical limits. The posterior segment of the fusiform gyrus and the inferior and medial temporal gyri composes it.

Currently, there are several techniques that can demonstrate brain networks. These techniques are grouped under the term “brain connectivity.” Fiber tractography, based on diffusion tensor imaging, demonstrates structural connectivity or direct connection of brain areas through associative tracts. Remote temporal correlation of the BOLD-signal history of a voxel or cluster, based on resting state sequences, demonstrates the functional connectivity through direct or indirect pathways.

Functional connectivity has been shown to be dynamic as it changes from task to task [45, 46] while structural connectivity is rather stable. Resting state-based functional connectivity represents a powerful tool to study and characterize different networks, but it has the limitation that it reflects the brain in a passive status and hinders important networks.

Recently, an alternative approach to study the brain connectivity has been proposed by Robinson et al. [47],* meta-analytic connectivity modeling*, or MACM. MACM allows examining task independent coactivation patterns of a specific anatomically defined brain region. MACM is based on automatic meta-analysis done by pooling coactivation patterns. The technique takes advantage of the Brainmap.org's repository of functional MRI studies [48, 49] and of a special software (Sleuth) provided by the same group, to find, filter, organize, plot, and export the peak coordinates for further statistical analysis. Sleuth provides a list of foci, in Talairach or MNI coordinates, each one representing the center of mass of a cluster of activation. The method takes the region of interest (for instance, a given Brodmann area), makes it the independent variable, and interrogates the database for studies showing activation of the chosen target. The query is easily filtered with different conditions (such as age, normal versus patients, type of paradigm, and domain of cognition). By pooling the data with these conditions, the tool provides a set of coactivations that can be statistically analyzed for significant commonality. As a final step, Activation Likelihood Estimation (ALE) [50, 51] can be performed using* GingerALE*; this is another software that is also provided by Brainmap; it generates the probability of an event to occur at voxel level across the studies. Areas of coactivation will show a network related to the function and domains selected as filter criteria. The nature of the analysis that is performed with hundreds to thousands of subjects signifies an improvement in generalizability and power but, overall, allows looking into global brain connectivity when the brain is at work, in contrast with functional connectivity that is mostly explored having the subject at rest (as in resting-state fMRI). Effective connectivity, another popular technique, may also look into brain connectivity at work, but it looks into specific task base related networks.

The aim of this work is to characterize the networks of language and visual perception in which BA37 is involved in normal samples, utilizing MACM. We aim to find possible commonality, divergences with findings from clinical, anatomical, and histological previously established knowledge. Although previous studies have indirectly analyzed in neurological samples the functionality of BA37 using functional MRI (fMRI) and diffusion tensor imaging (DTI) [52–54] their results have limited generalizability due to the methodologies (e.g., task-specific fMRI which is not generalizable and DTI which is influenced by deeply myelinated regions and fails where fibers cross) being employed [47]. Thus, strong empirical evidence is lacking as to how BA37 is functionally connected to the rest of the brain, despite the importance of this multimodal brain area in visual and cognitive processes. Recently Caspers et al. [55] using a meta-analytic connectivity modeling based on the Brainmap database observed that the fusiform gyrus is involved in different cognitive tasks, including object recognition, visual language perception, or visual attention.

It was hypothesized that BA37 represents an important cortical hub involving either language or visual processing depending on the specific cognitive computational load.

## 2. Materials and Methods

The DataBase of Brainmap (http://brainmap.org/) [48, 49, 56] was accessed on October 10, 2013, utilizing Sleuth 2. Sleuth is the software provided by Brainmap to query its database. Two meta-analyses were initially conducted (first level analysis). The first meta-analysis was intended to assess the language network in which BA37 is involved. The second meta-analysis was intended to assess the visual perception network in which BA37 is involved. A third meta-analysis (second level analysis) was then performed to assess contrasts and convergence between the two cognitive domains (language and visual perception). The anatomical localizations of the meta-analysis foci have been taken verbatim from the GingerALE output. All the studies were full brain coverage; a priori based studies were excluded. In an effort to avoid the effect of uncontrolled variables, clinical populations were excluded and only normal subjects entered in our study.

Noteworthy, BA37, or Brodmann's area 37, is defined internally in the application based on the MNI template (we did not use Talairach demon as a separate application). Therefore, the area of activation is provided automatically by the tool. All Brodmann's atlases are schematic segmentations of anatomical templates that are necessarily an approximate estimation of functional boundaries. Of note is the fact that BA37, as the vast majority of Brodmann's areas,* does not have overt anatomical boundaries*.

### 2.1. First Level Analysis

#### 2.1.1. Query 1: Language

The search conditions were (1) studies reporting BA37 activation; (2) studies using fMRI; (3) context: normal subjects; (4) activations: activation only; (5) handedness: right-handed subjects; (6) age 20–60 years; (7) domain: cognition; and (8) subdomain: language. Twenty papers with 28 suitable experiments with a total of 403 subjects and 413 foci were obtained (Table 1). Coactivation coordinates in MNI space were exported to text files.

ALE meta-analysis was then performed utilizing GingerALE. ALE maps were thresholded at *P* < 0.01 corrected for multiple comparisons and false discovery rate. Only clusters of 200 or more cubic mm were accepted as valid clusters. ALE results were overlaid onto an anatomical template suitable for MNI coordinates, also provided by Brainmap.org. For this purpose we utilized the Multi-Image Analysis GUI (Mango) (http://ric.uthscsa.edu/mango/) [90], Mosaics of 5 × 7 insets of transversal fusioned images were generated, utilizing a plugin of the same tool, selecting every other image, and starting on image number 10, and exported to a 2D jpg image.

#### 2.1.2. Query 2: Visual Perception

Search conditions were the same used in Study 1, except that instead of subdomain language, subdomain visual perception was entered. Thirteen papers with 20 suitable experiments, 130 subjects, and 185 loci were obtained (Table 2).

Subsequently, ALE meta-analysis was performed utilizing the same technique and settings described for the first meta-analysis.

### 2.2. Second Level Analysis

Contrast analysis of the two datasets was performed utilizing the GingerALE tool. For this purpose Language and Visual perception thresholded data images were uploaded into the software as Data Set 1 and Data Set 2, respectively. The foci coordinates of both domains were pooled in a single text file. Results were thresholded at 0.01 corrected for multiple comparisons with False Discovery Rate method. To correct for study sizes [91], GingerALE creates simulated data to find after many permutations of possible subtraction a voxelwise *P* value image, to show where the true data's values sit on the distribution of values in that voxel. ALE values are converted to *Z* scores to show the significance of the difference. Three outputs were obtained: (1) visual perception: perception contrast subtracted from Language contrast; (2) language contrast subtracted from visual perception contrast; and (3) conjunction of activations map.

The first output shows statistically significant activation in the network of language not present in the visual perception one. The second output shows the reverse, and the conjunction map shows areas in common between the networks.

## 3. Results

### 3.1. BA37 in Language

**Figure 1:**
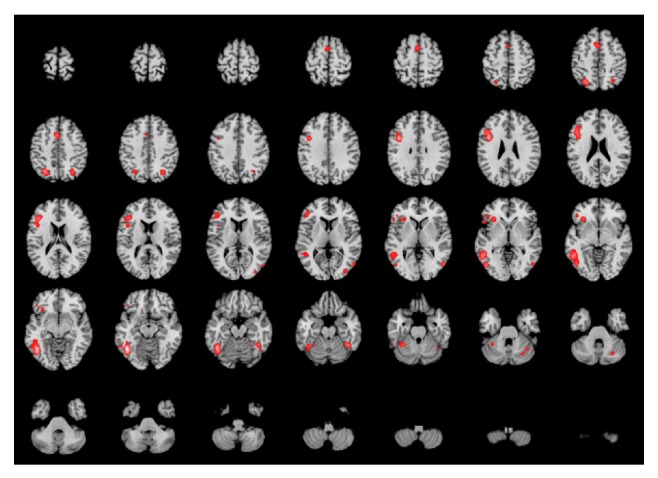
Language-related BA37's network. ALE results overlaid on an axial-T1 MRI MNI-template. Left hemisphere appears in the left side of the insets (neurological convention). ALE scores are color coded from red (lower scores) to white (higher scores). In addition to the left BA37 (middle and inferior temporal gyri in the convexity and fusiform gyrus in the basal aspect) that has the highest intensity, the following regions appear “activated”: left inferior frontal gyrus; bilateral superior and inferior parietal lobule (intraparietal sulcus); left SMA and posterior lobe of the cerebellum. There is also small activation of the right fusiform gyrus and right temporooccipital areas.

ALE score varied between 1.809E-20 and 0.0487 (maximum). For the threshold selected the minimum ALE value included was 0.0146.  Table 3 presents the main loci of BA37—connectivity related to language, by MACM. Twelve different clusters were found, 6 related to the left hemisphere and 6 to the right hemisphere. Significantly higher connectivity values, as represented by higher ALE scores, are located in the left hemisphere. BA37 presents significant connection with left inferior temporal lobe (BA20); left prefrontal cortex (BA9, BA46, BA45, and BA47), insula, bilateral precuneus (see limitations ahead), cerebellum, and occipital areas.  Figure 1 shows the areas of connectivity in a brain template.

### 3.2. BA37 in Visual Perception Functions

**Figure 2:**
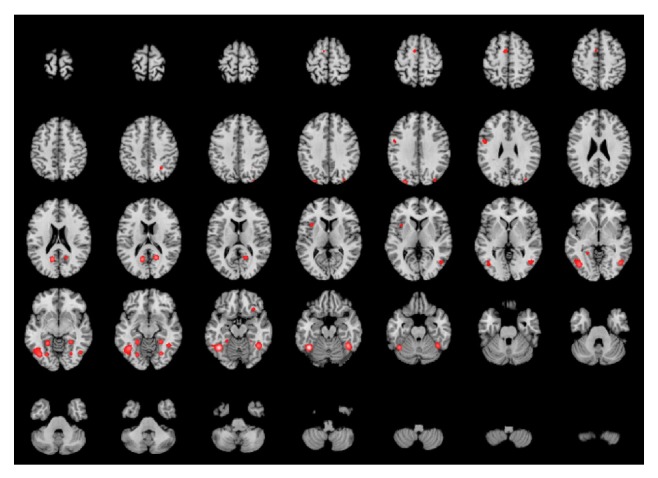
Visuospatial-related BA37's network. Same conventions and setting as described previously. The highest ALE scores are located on the left and right fusiform gyri (3–5 insets in fourth row) and parahippocampal and posterior cingulate gyrus (medial posterior activation in first two columns). Smaller and less intense activations appear in the left lateral premotor area, left SMA, left insula, and left inferior frontal gyrus. The intraparietal sulci activate bilaterally.

Table 4 and Figure 2 present the main loci of brain connectivity of BA37 by MACM for the second meta-analysis. Sixteen different clusters were found, 8 related to the left hemisphere and 8 to the right hemisphere. The main connections are within left and right BA37 and with the left visual association area (BA19). Significant connections are also observed with the cingulate gyrus (BA30), the medial aspects of the temporal lobe (parahippocampal area, BA36), and premotor cortex (BA6). A tiny cluster in right BA47 is also obtained. The connection found with the right anterior lobe of the cerebellum and culmen may be actually an artifact produced by the signal smoothing. The fusiform gyrus is adjacent to the culmen of the cerebellum, only separated by the tentorium. Since the clusters are the result of 3D spatial smoothing of the peaks of activation a “spread” to this structure should be expected. Maps of activation were automatically smoothed by the processing software. The analysis of the fused image seems to support this view (Figure 2).

### 3.3. Contrast Analysis

**Figure 3:**
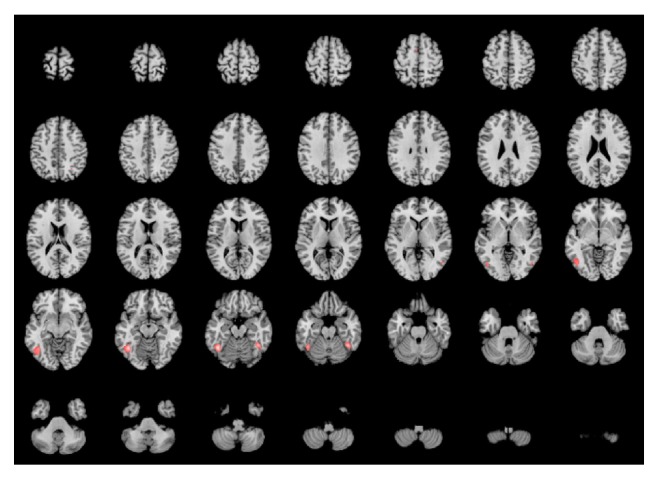
Conjunction analysis of BA37—language and visuospatial networks. Brain anatomical template and orientation are the same as priorly described. Intensities correspond to ALE scores. Color coding range from red (ALE score 0.01) to white (ALE max = 0.039). Main activations are mostly located in the fusiform gyri and adjacent lateral areas of the left side. A small cluster is observed in the left SMA (inset 5 in first row). This cluster however did not pass the volume threshold (vol = 48 cubic mm).

The subtraction of the language contrast minus the visual perception contrast (areas network specific for language involvement of BA37) produced two significant clusters. The largest cluster was localized in the lateral aspect of the left BA37 (center of mass MNI: −54, −45, 0); the smaller was localized in the left inferior frontal gyrus (center of mass MNI: −44, 17, 22) (BA45). The contrast of the visual perception contrast minus the language contrast (areas network-specific for visuospatial involvement of BA37) did not render any statistically significant differences (activation). The conjunction analysis revealed two clusters of commonality. The first centered at −45, −58, and 13, encompassing left BA37, BA19, and BA20 corresponding to the inferior temporal gyrus, middle and inferior occipital gyrus, and fusiform gyrus. The second cluster is centered at MNI 41, −49, and −20, encompassing right BA37 and BA20, corresponding to right fusiform gyrus. Both clusters list also the culmen of cerebellum that should be explained under the same bases already mentioned.  Figure 3 shows the statistically significant areas of commonality between the two domains.

## 4. Discussion

The purpose of the study was to analyze the brain networks of language and visual perception in which BA37 is involved in normal subjects. Our results may advance the understanding of the brain language systems as they suggest potential convergences with previously established neurological and neuropsychological findings.

The contribution of the paper revolves around the issue of understanding brain connectivity. Brain connectivity is still an evolving field, in which many questions are still unsolved. For example we need to understand why different techniques assessing brain connectivity yield distinct results. Structural connectivity and functional connectivity may show some similarities but also some discordant findings. A different perspective may shed lights to understand brain connectivity discordance, particularly if the new method explores the brain at work.

BA37 indeed is a complex and relatively large brain area, not only from the clinical (e.g., [5, 25]) but also from the functional perspective (e.g., [55, 92]), has been demonstrated to be involved in different cognitive abilities, including language and visual perception.

We have demonstrated two different connectivity networks related to BA37 utilizing the meta-analytic connectivity model. As expected these two networks are domain specific—one involving language and the second one including visual perception tasks. Noteworthy, our results are quite coincidental with those results reported by Caspers et al. [55].

### 4.1. Language

The differences in connectivity between these two domains deserve further analysis. The greatest connectivity of BA37 with BA20 in language tasks is not surprising. Functional neuroimaging studies suggest that BA20 participates in different language-related activities, such as lexical-semantic processing, metaphor comprehension, language comprehension and production, and selective attention to speech (see [24]). On the other hand BA37 is also significantly connected to the left prefrontal cortex (BA9 and BA46), which is involved in language control, verbal fluency, language generation, and verbal reasoning; this is the specific prefrontal area usually damaged in cases of transcortical motor (or dynamic) aphasia [20, 93]. So, left BA37 seemingly participates in a broader language system, related to complex language processing and understanding.

The significant connection of BA37 with the left insula is intriguing, even though it has been pointed out that the insula may have significant language and speech functions [94–97]. Interestingly, BA37 is connected to BA45 and BA47, areas that have been included in the so-called “Broca's complex” [98]. Another interesting connection is with the cingulate gyrus (BA32) involved in language initiative [16], as its pathology results in decreased spontaneous speech and mutism [99]. The connection to BA6 (the largest BA) is not surprising either; it has been established that BA6 participates in diverse language functions, including language processing, language switching, speech perception, phonological processing, object naming, word retrieval, and lexical decision on words and pseudowords (see [24]). Considering the involvement of BA37 in visual perception the connections with BA19 (visual association area) seem understandable; it emphasizes that BA37 has not only language but also visuoperceptual functions. The connection to left BA7 points to an involvement of the circuitry in verbal working memory [100]. Strikingly, BA37 does not seem to be directly connected with BA44, BA21, and BA22—the most classical expressive and receptive language areas. However, it is prominently connected to BA 45 (inferior frontal gyrus,* pars triangularis*, currently regarded as the anterior segment of Broca's area) and BA20 (inferior temporal lobe) which has an evident participation in language understanding and processing: lexicosemantic analysis, metaphor comprehension, language comprehension and production, and selective attention to speech [24].

It has to be noted that quite diverse language tests were used in the different studies entered in our meta-analysis, including language production (e.g., overtly or covertly generate words, category fluency), naming (e.g., naming objects, action naming), language understanding (e.g., semantic decision on words, word association), repetition (e.g., repetition emotional neutral words), and reading (e.g., word reading). This heterogeneity in the verbal tasks that were used can partially account for the extended net of brain interconnections that was found. However, regardless of the heterogeneity, the core language function of BA37 seemingly refers to language semantics [101].

Noteworthy, for language tasks most of the activation clusters were located in the left hemisphere, whereas for visual perceptual tasks a more bilateral—particularly posterior—activation was observed. This of course is congruent with left language lateralization, whereas visual perception has a more bilateral organization; supposedly the posterior right hemisphere has a crucial role in visual recognition, whereas for visual naming posterior left hemisphere areas are involved [102–106].

The right hemisphere connections are of special interest. First, both left and right BA37 are interconnected, and hence some coordinated activity between both areas has to be assumed. But also a significant connection is found with secondary visual areas of the right hemisphere, emphasizing the involvement of BA37 in visual-perceptual functions. The connection of BA37 with the right parietal precuneus (BA7) is suggestive of some involvement in spatial orientation and visual attention, considering that the pathology in this cortical area is usually associated with significant spatial orientation disturbances and left-hemispatial neglect [5, 102, 107].

### 4.2. Visual Perception

With respect to the visual perception network, the connectivity of BA37 with the parahippocampal gyrus suggests some involvement in memory processing as indeed has been previously reported [108, 109]. Noteworthy, five out of the 16 clusters involve the occipital lobe, emphasizing the participation of BA37 in visual-perceptual processing circuits. Cluster number 14 points out that BA37 maintains significant connection with the insular region related to a diversity of functions. fMRI studies have demonstrated that the insula participates not only in motor and sensory processes, but also in pain, temperature, touch, olfaction, taste, language, memory, emotion, and so forth (see [24]).

In this second meta-analysis (visual-spatial), two significant connections with the frontal lobe were found: with the left medial frontal gyrus (BA6) and with the right inferior frontal lobe (BA47). Even though the specific functions of right BA47 are not sufficiently clear yet, it has been reported that during behavioral inhibition, enhanced activations are observed in the right orbitofrontal cortex (BA47) [110]. Another interesting connection with right parietal precuneus (BA7) as mentioned above, departing from clinical observations it can be assumed that the right parietal precuneus (BA7) is involved in visuoconstructive abilities, spatial representation, and visual-spatial attention [5, 102, 107]; connections between right BA7 and BA37 emphasize the involvement of BA37 (right) in visuoconstructive abilities, such as drawing and spatial attention.

### 4.3. Common Connection

It is noteworthy to mention that in spite of BA37 being a rather extensive area, the functional segregation of at least the two domains explored here does not seem to be related to any existent local cortical landmark. Indeed, two of the most significant peaks of the first clusters in both ALE analyses are in close proximity. The first clusters (L-fusiform gyrus) have a Euclidian distance of 6.9 mm, and the second (L-occipital fusiform/left occipitotemporal gyrus) −7.2 mm. Moreover, the conjunction analysis demonstrated the overlapping of the areas, pointing to a true multimodal function of the BA37. This is not surprising considering that BA37 seems to have two subregions separated by histological heterogeneity that could explain and hold functional multimodality. BA37's peripheral parts are transitional between neocortex and allocortex, similar to the bordering auditory and visual areas. On the other hand, BA37's central part (the “nucleus”) is typical neocortex, the most recent evolved of the human cortex [111]. Linguistic categorization of objects (naming) resides in this new type of cortex (i.e., linguistic function) [112].

The connectivity of BA37 with BA45 (*Pars triangularis*) probably points to a ventral pathway of language, as BA45 has been found to serve as a rostral terminus to the termed extreme capsule pathway in DTI studies [113]. This pathway is coincidental with the path of the inferior occipitofrontal fasciculus that has been found to produce semantic paraphasias when disrupted with intraoperatory deep electrical pulses [114]. Interestingly, linguistic inferences alone recruit left perisylvian regions of linguistic competence, including BA37, 44, and 45 [44] as well as frontal regions (BA6, 9, 46, and 47); this finding suggests that the semantic aspects of linguistic processing mediated by BA37 is relevant in complex thought processes. The specific connectivity between BA37 and 46 (dorsolateral prefrontal cortex) is most likely related to executive functions (i.e., executive control through language) including abstraction and complex thinking.

The participation of BA37 in both language and visual perceptual abilities supports the multimodality of this area. As a matter of fact, a segregation of different segments of the fusiform gyrus has been observed. Caspers et al. [55] refer to two distinct cytoarchitectonic areas, FG1 and FG2; FG1 appears as a transitional area between early and higher visual cortex and FG2 as a higher-order one. FG2 is furthermore lateralized and is associated to the visual language processing in the left hemisphere. It has also been suggested a fusiform face area (FFA) in the right hemisphere [115] and a visual word-form area (VWFA) in the left [116], indicating the multimodality of BA37. Interestingly, reading systematically activates the left lateral occipitotemporal sulcus, frequently referred to as VWFA. Furthermore, this observation is reproducible across individuals/scripts, specific to reading-specific processes, and partially selective for written strings relative to other categories (e.g., line drawings); it is also well known that its lesion causes pure alexia (inability to recognize words) [117].

It has to be considered the possibility that the activated sites in BA37 in language tasks were not identical with those activated in visual processing tasks; BA37 is a relatively large area, and in the contrast of language minus visual perception residual clusters in left BA37 were found. However, seemingly VWFA is included in the clusters, suggesting that BA37 clearly participates in language-visual perception associations.

Damage in BA37 has been traditionally accepted to be associated with significant word-finding difficulties (anomia) (e.g., [20, 118–120]), impaired naming of pictures, and relatively preserved word comprehension [120, 121]. This word-finding defect associated with temporal-occipital damage has been further proposed to correspond to a particular aphasia syndrome named anomic aphasia [5], nominal aphasia [122], or amnesic aphasia [20] or sometimes simply regarded as a subtype of what is termed transcortical (or extrasylvian) sensory aphasia [14, 16, 123].

In anomic (or nominal or amnesic) aphasia, semantic paraphasias are abundant. As a matter of fact, the damage in this area results in the highest amount of semantic paraphasias observed in cases of brain pathology [16, 25, 124, 125]. Because of the location of the pathology (temporooccipital), it is not surprising that visual agnosic defects can also be found in these patients; indeed, they present a significant impairment in revisualizing for him/herself the meaning of the words (i.e., how a “book,” or a “dog” or whatever noun looks like) [126]. Thus, anomic or nominal or amnesic aphasia can be interpreted as a language defect at the level of the semantics of the words [14]: the patient fails in associating the words of the vocabulary (e.g.,* table*,* chair*) with their visual meaning (revisualizing the meaning of the word* table*,* chair*, etc.); and conversely, the visual representations (images or pictures of* tables*,* chairs*, etc.) do not evoke a specific word but a diversity of semantically related words (such as* desk*,* bed*, and* sofa*).

de Renzi and Saetti [127] have proposed that “optic aphasia” (a name frequently used as synonymous of visual anomia) and associative visual agnosia (i.e., the impaired visual recognition or inability to assign meaning to a stimulus when early stage perceptual processing is preserved) reflect the impaired access of structured representations of semantics. The anatomical bases of the two syndromes may be very similar (or even coincidental), and hence, optic aphasia can to some extent be regarded as an associative visual agnosia. As a matter of fact, optic aphasia could be interpreted as a disconnection syndrome between visual perception and semantic associations. In associative visual agnosia the patients correctly recognize the primary visual characteristics of the objects but fail in recognizing what they are; copying figures is correct, demonstrating that the patient successfully recognizes line orientations, curvatures, spatial distribution, size, and so forth [103, 128]. Conversely, in apperceptive visual agnosia patients cannot copy figures because the early-level perceptual processing is impaired (i.e., the ability to recognize the primary characteristics of the visual stimuli, such as lines, spatial location, and relationship among the different elements).

In summary, left BA37 damage has been associated with a language disturbance characterized by word-finding difficulties for the visually presented information (visual anomia); additionally, these patients present some visual-perceptual disturbances, similar to an apperceptive visual agnosia. Right BA37 pathology usually results in prosopagnosia and visoconstructive disturbances.

We have to be aware that “semantic” language function is an extended language function including different elements, such as memory, visual representations, and even executive (frontal) functions [129]. BA37 is only related to language visual representation, a particular aspect of language semantics.

Whereas language semantics has been partially related to left BA37, right BA37 has been associated with complex visual functions, such as face recognition and structural judgment of familiar objects. It is well known that prosopagnosia (acquired inability to recognize faces) is the result of brain pathology involving the right fusiform gyrus (temporal-occipital) or both fusiform gyri [130–133].

Disturbances in drawing (constructional apraxia or simply visuoconstructive disorder) are also observed in cases of right hemisphere pathology frequently involving the right BA37 [5, 102, 134, 135]. Constructional apraxia is usually defined as an inability or difficulty to assemble, build, or draw objects and has been traditionally considered as a major right hemisphere syndrome although a milder syndrome is sometimes observed after damage to the left hemisphere [136]. Noteworthy, the mental imaging of drawing has been associated with activations of both right and left BA37 [137].

Jouen et al. [129] tested the hypothesis that comprehension of human events engages an extended semantic representation system, independent of the input modality (sentence versus picture). They examined brain activation and connectivity (fMRI and DTI) in 19 subjects who read sentences and viewed pictures depicting everyday events. A common frontotemporoparietal network including the middle and inferior frontal gyri, the parahippocampal-retrosplenial complex, the anterior and middle temporal gyri, the inferior parietal lobe and in particular the temporoparietal cortex was found. DTI tractography revealed a multicomponent network involving the temporal pole, the ventral frontal pole, and premotor cortex. The authors concluded that “meaning” network includes semantic memory, embodied simulation, and visuospatial scene representation. These findings are congruent with our results suggesting that the posterior temporal and temporal-occipital area are involved in the visual representation of the meanings of the words.

It is important to emphasize that we do not know if current results are applicable to bi/multilingual individuals. The use of two or more different languages can result in some reorganization of the language in the brain [138, 139] but this is a point for future research.

Some limitations of the current meta-analytic study should be addressed by future research. Our thresholds may have been quite conservative leaving out small areas of coactivation and limiting the discrimination of commonalities and differences in the two networks (i.e., language and visual) in which BA37 has been associated with. Some limitations are inherent to the postprocessing software (GingerALE) that yields an automatic labeling of the activation clusters. These limitations are insurmountable for the authors. The ALE report lists activations in both precuneus areas (BA7 and BA19). Yet, the coregistered rendition of the activation maps does not show such activation. Instead, there is bilateral activation in both banks (superior and inferior) of the intraparietal sulcus that may explain BA7 and BA19 localization. This activation is quite concordant with prior findings in language function. A correction to this shortcoming has been done in the figure captions. In addition, our study, like most meta-analysis, has the limitation of the heterogeneity of the pooled tasks, methods, and individuals. Despite these limitations, this study presents a connectivity model using BA37 as independent variable and a spectrum of coactivated areas as dependent variables. The results found here are quite consistent with clinical findings and highly supported by structural connectivity findings. Although MACM is still new, previous studies have already validated the use of this technique to analyze the connectivity of other brain areas [47].

## 5. Conclusions

Taken together the clinical and functional information about BA37 make it clear that left BA37 participates in at least two distinct functional domains—visual recognition (perception) of external objects and associating the visual perception with a word (newer BA37 segment). These two domains are related to two distinct networks in which BA37 overlaps. Within the first network, BA37 projects to BA46 (prefrontal area involved in executive functions including abstraction and complex thinking) and BA9 and BA45 (involved in word generation, semantic categorization, and metaphor comprehension). In the second network BA37 projects to parahippocampal and posterior cingulate regions related to memory; BA19 related to visual processing and BA6 and BA47 of executive control.

Finally, it should be emphasized that current results are congruent with our existing clinical (e.g., [5, 25, 107]) and functional (e.g., [55, 92]) knowledge on the role of BA37 in cognition.

## Figures and Tables

**Table 1:** Primary studies of language-related paradigms included in the first meta-analysis.

Publication	Paradigm	*n*	Foci
Binder et al., 1996 [57]	Passive listening words	12	6

Booth et al., 2002 [58, 59]	Auditory rhyming	13	7
	Visual meaning-rhyming	13	3

Palmer et al., 2001 [60]	Overtly or covertly generate words	10	26

Crosson et al., 1999 [61]	Repetition emotional neutral words	17	7

Devlin et al., 2003 [62]	Semantic + phonological	12	26
	Phonological > semantic	12	34

van Turennout et al., 2003 [63]	Naming novel, repeated objects	10	11

Binder et al., 2003 [64]	Stimuli were words or nonwords	24	26

Booth et al., 2002 [58, 59]	Visual words spelling	13	9

Gold and Buckner, 2002 [65]	Semantic decision on words	24	3

Mechelli et al., 2006 [66]	Naming black and white objects	12	22

Saccuman et al., 2006 [67]	Word class-semantic reference	13	18

Pihlajamäki et al., 2000 [68]	Category fluency	14	9

Jobard et al., 2007 [69]	Word reading	10	12

Damasio et al., 2001 [70]	Action tool word retrieval	20	1
	Concrete entities	20	5

Simmons et al., 2008 [71]	Word association	10	32
	Property generation	10	26

Davis et al., 2004 [72]	All words versus letter strings	12	9
	Verbs versus noun	12	1

Liljeström et al., 2008 [73]	Action naming	15	30
	Object naming action images	15	24
	Object naming simple images	15	12

Chee et al., 2003 [74]	Low and high frequency	12	7
	Low and high frequency	12	10

Sabsevitz et al., 2005 [75]	Concrete > abstract nouns	28	26

Bedny and Thompson-Schill, 2006 [76]	Nonwords > words	13	11

**Table 2:** Primary studies of visual perception paradigms included in the second meta-analysis.

Publication	Paradigm	*n*	Foci
Hasson et al., 2002 [77]	Faces > letters and buildings	13	4

Vaidya et al., 2002 [78]	Pictures versus words	8	2

Vandenberghe et al., 2001 [79]	Spatial shifting	12	17

Shen et al., 1999 [80]	Spatial recognition > visual recognition	9	12

Kesler-West et al., 2001 [81]	Facial emotion processing	21	17

Vingerhoets et al., 2002 [82]	Mental rotation	12	3

Vanrie et al., 2002 [83]	Mental rotation	6	6

Wraga et al., 2005 [84]	Imagined rotations	11	7

Giesbrecht et al., 2003 [85]	Foveal, location spatial attention	10	12
	Peripheral, color	10	12
	Peripheral, color > location	10	4

Dolan et al., 1996 [86]	Matching faces	8	4

Vuilleumier et al., 2001 [87]	Attention, faces > houses	12	3
	Attention, houses > faces	12	7

Creem-Regehr and Lee, 2005 [88]	Representation of objects	12	10
	Shapes > scrambled shapes	12	1
	Tools, imagined grasping	12	15
	Imagined grasping	12	13

Blonder et al., 2004 [89]	Brain responses to faces	14	10
	Images versus human faces	14	14
	Dog faces versus house	14	12

**Table 3:** Main loci of brain connectivity of BA37 in language tasks by meta-analytic connectivity modeling (MACM).

Region (BA)	*x*	*y*	*z*	ALE	Volume (mm^3^)
**Cluster number 1**					
L fusiform gyrus (37)	−46	−58	−14	0.048781	9,568
L subgyral grey matter (37)	−54	−48	−4	0.03231	
L occipital-temporal gyrus (37)	−48	−70	−2	0.028959	
L inferior temporal lobe (20)	−60	−52	−14	0.017783	
**Cluster number 2**					
L inferior frontal gyrus (9)	−42	8	28	0.041418	7,552
L middle frontal gyrus (9)	−48	20	24	0.028233	
L middle frontal gyrus (46)	−44	28	18	0.027926	
L inferior frontal gyrus (46)	−46	38	8	0.022982	
L insula (13)	−46	14	14	0.022624	
L inferior frontal gyrus (45)	−46	34	−4	0.019394	
L middle frontal gyrus (47)	−46	36	−12	0.016915	
**Cluster number 3**					
L middle frontal gyrus (32)	−4	14	48	0.027447	2,184
L middle frontal gyrus (6)	−2	8	60	0.023249	
**Cluster number 4**					
L precuneus (19)	−30	−64	48	0.033621	1,872
**Cluster number 5**					
L inferior frontal gyrus (47)	−34	28	−6	0.023995	1,336
**Cluster number 6**					
R precuneus (7)	30	−66	44	0.025861	1,304
**Cluster number 7**					
R fusiform gyrus (37)	46	−60	−18	0.01576	1,168
**Cluster number 8**					
R occipital temporal gyrus (37)	52	−68	2	0.026111	824
**Cluster number 9**					
R posterior lobe of the cerebellum and pyramid of vermix	30	−68	−32	0.020669	424
**Cluster number 10**					
R middle occipital gyrus (18)	34	−86	8	0.022077	400
**Cluster number 11**					
R anterior lobe	40	−58	−30	0.020532	288
**Cluster number 12**					
L inferior frontal gyrus	−52	20	−2	0.017183	240

**Table 4:** Main loci of brain connectivity of BA37 in visual perceptual tasks by meta-analytic connectivity modeling (MACM).

Region (BA)	*x*	*y*	*z*	ALE	Volume (mm^3^)
**Cluster number 1**					
L fusiform gyrus (37)	−42	−54	−18	0.043706	5,792
L occipital fusiform gyrus (19)	−42	−70	−6	0.025872	
L Ffsiform gyrus (37)	−56	−52	−16	0.013379	
**Cluster number 2**					
R ant lobe cerebellum culmen	40	−50	−20	0.031403	2,176
**Cluster number 3**					
R occipital temporal lobe (37)	46	−66	−4	0.023151	1,232
**Cluster number 4**					
L parahippocampal gyrus (36)	−24	−46	−8	0.024606	1,000
**Cluster number 5**					
L posterior cingulate gyrus (30)	−14	−58	18	0.031237	968
**Cluster number 6**					
R posterior cingulate gyrus (30)	16	−54	16	0.028388	856
**Cluster number 7**					
R parahippocampal gyrus (36)	24	−42	−10	0.023156	688
**Cluster number 8**					
L medial frontal gyrus (6)	−8	0	56	0.017237	616
**Cluster number 9**					
L precentral gyrus (4)	−52	0	28	0.016724	512
**Cluster number 10**					
L occipital fusiform gyrus (19)	−26	−70	−10	0.022296	480
**Cluster number 11**					
L cuneus (19)	−30	−86	34	0.028235	440
**Cluster number 12**					
R occipital fusiform (19)	26	−68	−10	0.021505	416
**Cluster number 13**					
R cuneus (19)	32	−82	38	0.019212	400
R middle occipital gyrus (19)	36	−80	28	0.013487	
**Cluster number 14**					
L insula (13)	−38	14	6	0.016739	376
**Cluster number 15**					
R inferior frontal lobe (47)	30	28	−16	0.015931	240
**Cluster number 16**					
R precuneus (7)	26	−56	44	0.01558	
